# Supplementary figures and images for: Cross-tissue coordination between SLC nucleoside transporters regulates reproduction in Caenorhabditis elegans
Source: PLoS Genet. 2025 May 30;21(5):e1011425. doi: 10.1371/journal.pgen.1011425 (PMC12151481; doi:10.1371/journal.pgen.1011425)

A

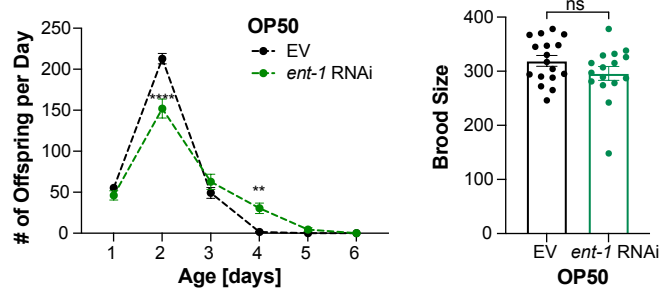

F

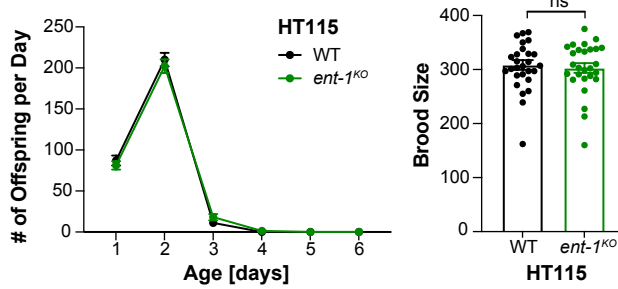

C

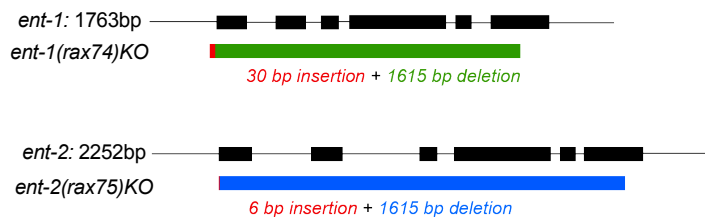

H

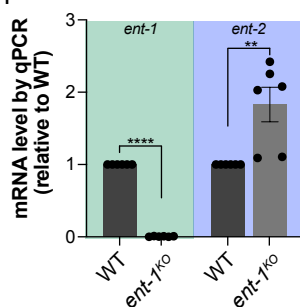

I

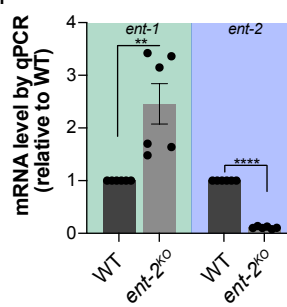

D

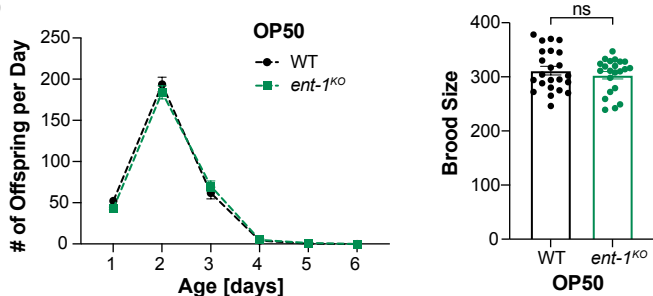

J

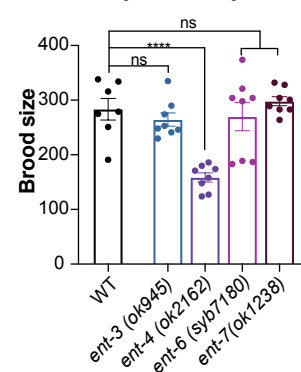

E

Supplement: S1 Fig — (A) RNAi knockdown of ent-1 on OP50 bacteria alters the daily progeny number compared to the EV control. n = 17(EV), n = 16(ent-1 RNAi). (B) RNAi knockdown of ent-2 on OP50 bacteria alters the daily progeny number compared to the EV control. n = 17(EV), n = 16(ent-2 RNAi). (A) (C) Genome illustrations of ent-1KO and ent-2KO mutants. (D) ent-1KO mutants on OP50 bacteria show no significant alteration of daily progeny number or brood size compared to wild type (WT) worms. n = 23(N2), n = 22(ent-1KO). (E) ent-2KO mutants on OP50 bacteria show no significant alteration of daily progeny number or brood size compared to WT. n = 18(N2), n = 18(ent-2KO). (F) ent-1KO mutants on HT115 bacteria show no significant alteration of daily progeny number or brood size compared to WT. n = 28(N2), n = 27(ent-1KO). (G) ent-2KO mutants on HT115 bacteria show no significant alteration of daily progeny number or brood size compared to WT. n = 22(N2), n = 22(ent-2KO). (H) (I) RT-qPCR analysis shows that the ent-2 mRNA level is upregulated in the ent-1KO mutant (H), and the ent-1 mRNA level is elevated in ent-2KO (I). n = 6 biologically independent samples in each condition. (J) The mutants of ent-3, ent-5, ent-6, or ent-7 display normal brood size compared to wildtype control, while ent-4(ok2161) has reduced brood size. Statistic: *p<0.05, **p<0.01, ***p<0.001, ****p<0.0001, ns p>0.05. Student’s t-test (unpaired, two-tailed) was applied for the brood size graphs in A, B, D, E, F and G, and RT-qPCR results in H and I. Two-way ANOVA with Bonferroni’s post hoc test for the daily progeny graphs in A, B, D, E, F and G. One-way ANOVA with Holm–Sidak correction for J. Data are shown as mean ± S.E.M. from at least three independent biological replicates, each with 5–10 animals, except (J) which is performed with two independent biological replicates, each with around 4animals. The “n” values represent the total number of animals across replicates. (PDF) [file pgen.1011425.s001.pdf]

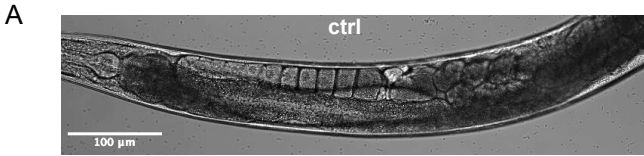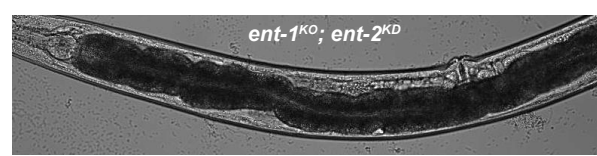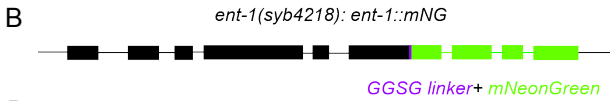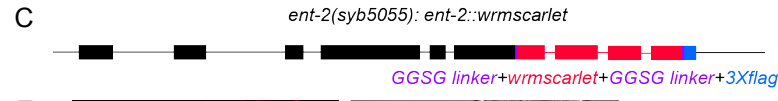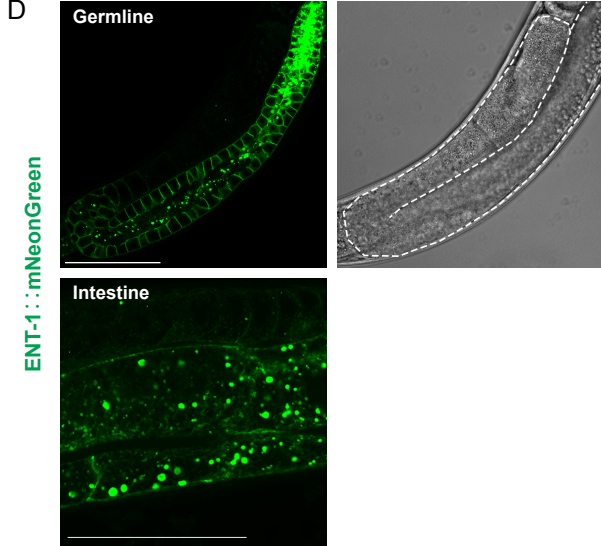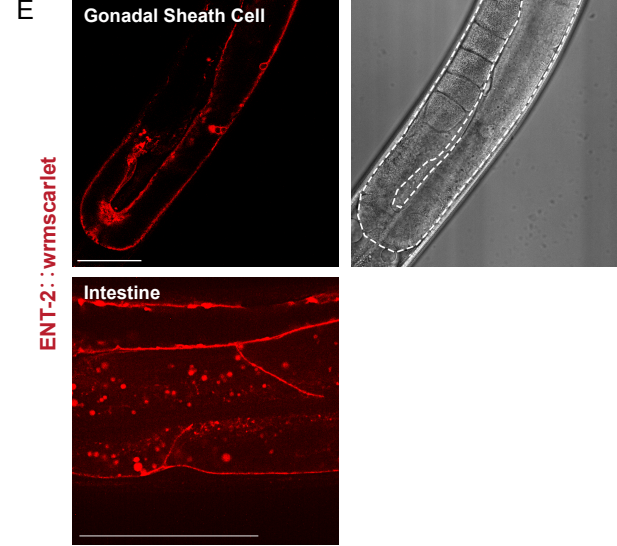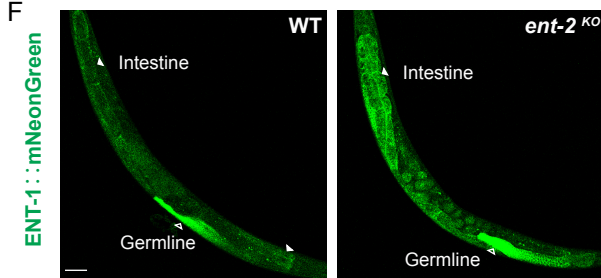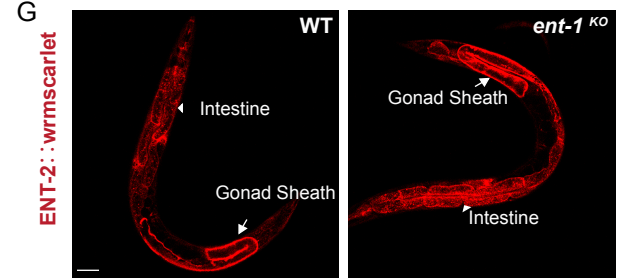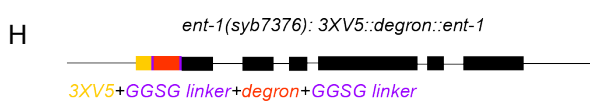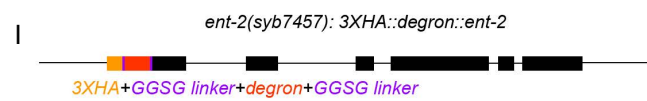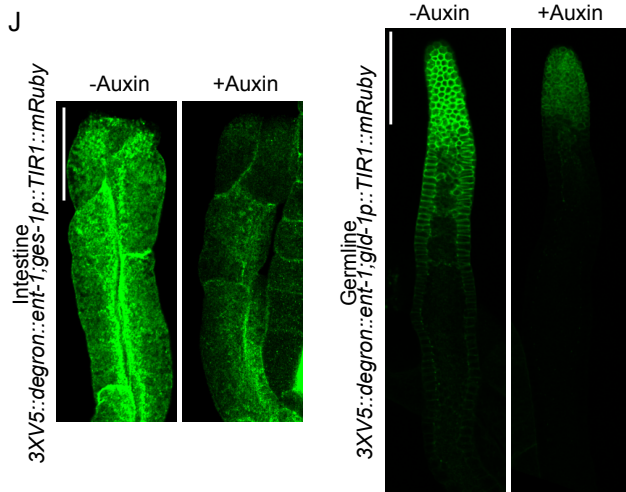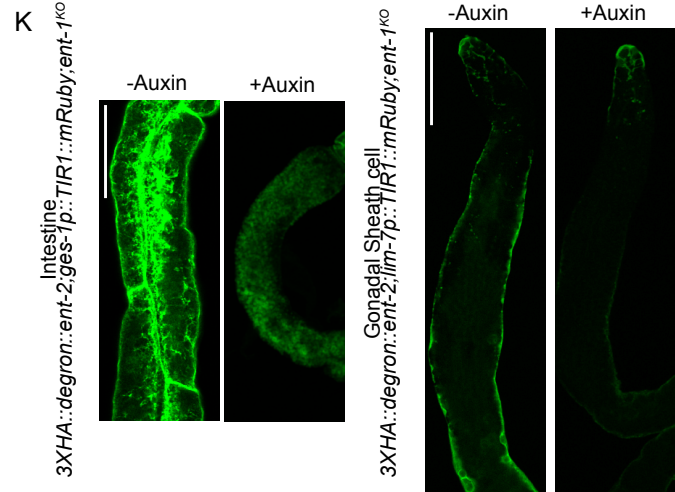

Supplement: S2 Fig — (A) Bright-field images show sterile ent-1KO; ent-2KD mutants and their controls. Scale bar: 100 µm. (B, C) Diagrams showing CRISPR knock-in strains: ent-1::mNeonGreen (B), ent-2::wrmscarlet (C). (D, E) Endogenous localization of ENT-1::mNeonGreen in the germline and intestine (D) and ENT-2::wrmscarlet in gonadal sheath cells and the intestine (E). Scale bar: 50 µm. (F) Images of ent-1::mNeonGreen in WT and ent-2KO. Scale bar: 50 µm. (G) Images of ent-2::wrmScarlet in WT and ent-1KO. Scale bar: 50 µm. (H) (I) Diagrams showing CRISPR knock-in strains: degron::ent-1 (H) and degron::ent-2 (I). (J) (K) Immunostaining results show the efficiency of tissue-specific degron-mediated protein degradation induced by the Auxin treatment in the degron::ent-1 strain (J) and in the degron::ent-2 strain (K). Scale bar: 50 µm. (PDF) [file pgen.1011425.s002.pdf]

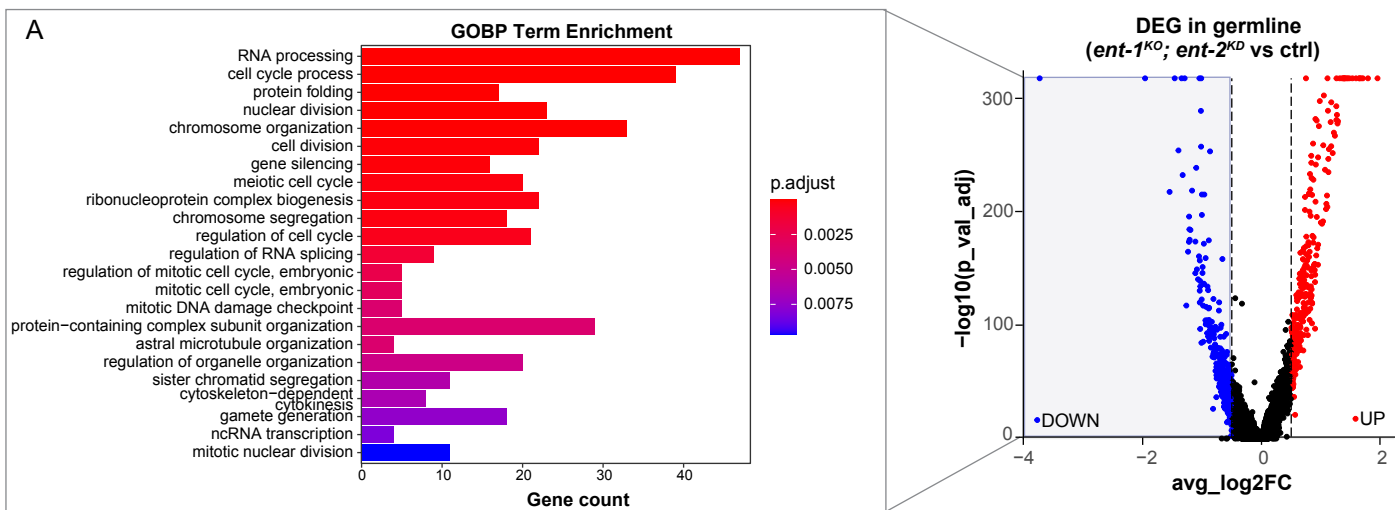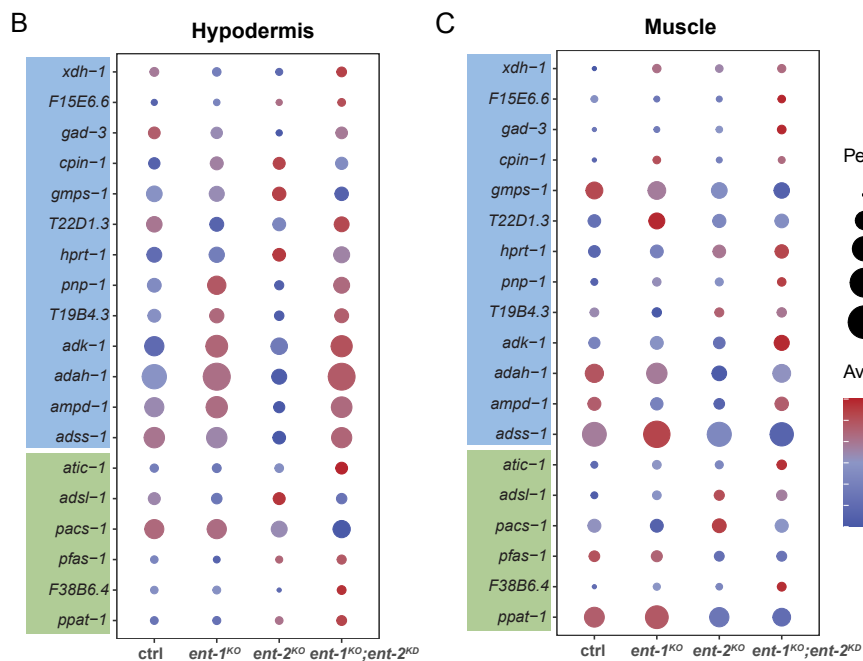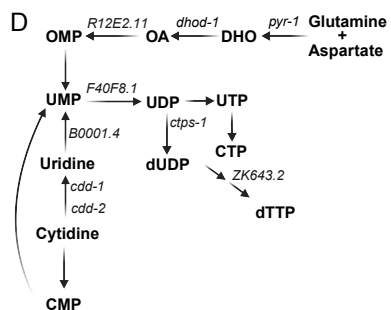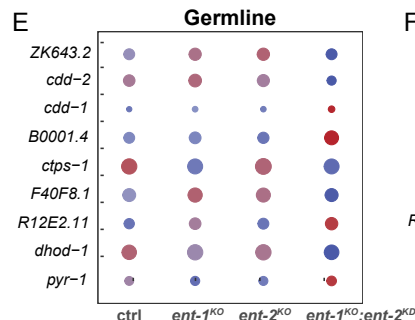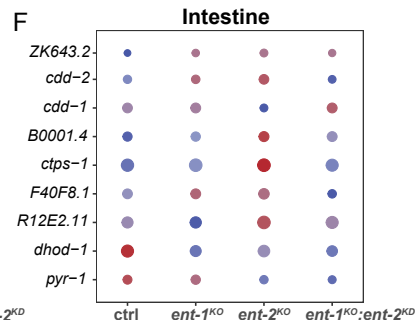

Supplement: S3 Fig — (A) Gene ontology enrichment analysis of biological processes was performed on the down-regulated expressed genes in the germline of ent-1KO; ent-2KD worms compared to their controls, revealing pathways related to cell cycle and division are overrepresented. (B) (C) Dotplots show the relative expression levels of genes that are associated with purine metabolism of control, ent-1KO, ent-2KO, and ent-1KO; ent-2KD animals in the hypodermis (B) and muscle (C). (D) Schematic illustration of C. elegans homologous genes involved in pyrimidine metabolism pathways. (E) (F) Dot plots show the relative expression levels of genes associated with pyrimidine metabolism in the germline (E) and intestine (F). (PDF) [file pgen.1011425.s003.pdf]
