# Supplementary material for: Cross-tissue coordination between SLC nucleoside transporters regulates reproduction in Caenorhabditis elegans
Source: PLoS Genet. 2025 May 30;21(5):e1011425. doi: 10.1371/journal.pgen.1011425 (PMC12151481; doi:10.1371/journal.pgen.1011425)
Supplement: Attachment — Submitted filename: point_by_point.pdf [file pgen.1011425.s005.pdf]

Point-by-point responses:

Reviewer #1: In this manuscript, Guan et al use sophisticated genetic methods to determine the tissue-specific requirements of the solute/nucleoside transporters ENT-1 and ENT-2 in the germline and intestine, respectively. They propose a model whereby nucleosides are release from the intestine via ENT-2 and subsequently taken up by the germ cells via ENT-1, and that this is required for proper reproductive health. Using snRNA-seq and metabolomics, they found that the guanosine was the primary metabolite that was exported by ENT-2 from the intestine and taken up by germ cells via ENT-1, and that this was essential for proper reproduction. The manuscript is very well-written, extremely easy to read, and the data is very strong. Overall, I believe the manuscript even in its current state is deserving of publication in PLoS Genetics and only have very minor comments, which I believe can strengthen the manuscript, but is certainly not essential.

Response: We appreciate the reviewer finding our paper well-written and recommending it for publication in PLoS Genetics.

1. The authors argue that ent-1 and ent-2 loss of function (lof) have much milder phenotypes than a double lof due to the compensation by the upregulation of the other transporter. However, this is inconsistent with a model whereby ENT-1 functions solely in the germline and ENT-2 functions solely in the intestine. Is it possible that ENT-1 primarily functions in the germline, but has the capacity to also function as a nucleoside exporter in the intestine when ENT-2 is lost? Similar for ENT-1?

Response: We appreciate the reviewer's suggestion that ENT-1 may function in the intestine when ENT-2 is lost, or *vice versa*. However, our results do not support this possibility.

1) In the *ent-2*^{KO} background, we did observe the up-regulation of *ent-1* in the intestine and the germline (**S2F Fig**). However, only germline-specific knockdown of ENT-1 resulted in reproductive defects with *ent-2*^{KO}, while intestine-specific knockdown of ENT-1 has no such effect (**Fig 2G, 2H**). Furthermore, the intestine-specific restoration of *ent-1* expression in *ent-1*^{KO}*; ent-2*^{KD} did not rescue the sterile phenotype (**Fig 2C**). These results do not support the contribution of intestinal ENT-1 to maintain reproduction upon the loss of ENT-2.

2) In the *ent-1*^{KO} background, we only observed the increase of ENT-2 in the intestine and somatic gonad, but not in the germline (**S2G Fig**). Moreover, only intestine-specific knockdown of ENT-2 resulted in reproductive defects with *ent-1*^{KO} (**Fig 2I**). These results do not support that ENT-2 functions in the germline upon the loss of ENT-1.

Based on our current findings, we concluded that the sterile phenotype observed in the double mutant results from the combined roles of ENT-1 in the germline and ENT-2 in the intestine. Mutating both genes disrupts guanosine export from the intestine to the pseudocoelom and its subsequent uptake into the germline, leading to an insufficient guanosine level in the germline

to support reproduction. We would like to clarify that guanosine in the pseudocoelom can be exported from multiple somatic tissues, not just the intestine. In the ENT-2 single mutant, although the guanosine export from the intestine is decreased, ENT-1 can still import some guanosine into the germline from the pseudocoelom. In the ENT-1 single mutant, the guanosine level in the pseudocoelom remains high (maybe even higher), and other transport mechanisms may compensate for the loss of ENT-1 and import some guanosine into the germline to support reproduction. As a result, the single mutant exhibited much milder phenotypes. In the revised manuscript, we have extended discussions regarding the possible compensatory mechanisms to sustain reproduction in the *ent* single mutants.

2. Since the authors have made reporters for both lines, it might be useful to see whether the increase in ENT-1 upon ent-2 lof occurs in both the germline and intestine, and the same for ENT-2 upon ent-1 lof. Minimally, the authors can analyze the snRNAseq data for this information and then speculate on something in the discussion about this conundrum.

Response: We thank the reviewer for the suggestion. We have obtained images of *ent-1::mNeonGreen; ent-2 KO* and *ent-2::wrmScarlet; ent-1 KO*, which demonstrate that ENT-1 protein expression is upregulated in both the intestine and germline under the *ent-2* single mutant background, while ENT-2 protein levels are upregulated in both the intestine and somatic gonad under the *ent-1* single mutant background. Additional discussions regarding the compensatory mechanisms in the *ent* single mutants are included in the revised manuscript.

3. The discussion seems very short. I feel several things can be expanded upon. For example, why do the authors believe that the other ENTs are not required for reproduction? Why do the authors believe that ENT-1 and ENT-2 are not required for development, despite nucleosides regulation being very important for cell division as they say in their introduction? How does the work fit in with other models of reproduction? Are these transporters essential in higher eukaryotic systems as well?

Response: We thank the reviewer for the suggestion to expand the discussion.

1) We have included additional experiments and discussions regarding the potential roles of other *ent* genes in reproduction. Since RNAi used in the initial ENT gene screening might have masked deleterious effects of *ent* genes beyond *ent-1* and *ent-2*, we measured brood sizes in the loss-of-function mutants of *ent-3 (ok945)*, *ent-4(ok2161)*, *ent-6 (syb7180)*, and *ent-7 (ok1238)* (noting that an *ent-5* mutant is currently unavailable). Our analysis revealed that the *ent-4* deletion mutant displayed a significantly reduced brood size compared to the wild type (**S1J Fig**).

Interestingly, prior research (PMID: 38991033) (Yanagi et al., 2024) indicates that ENT-4 is specifically localized to the apical membrane of intestinal cells, suggesting that it may mediate the uptake of nucleosides from the diet to regulate reproduction. In contrast, ENT-2 localizes to the basolateral side of the intestine, indicating a role in exporting nucleosides from the intestine to the pseudocoelom. Taken together, *ent* family genes might support reproduction in

a coordinated model: ENT-4 facilitates dietary nucleoside uptake into the intestine, ENT-2 exports nucleosides from the intestine to the pseudocoelom, and ENT-1 imports nucleosides from the pseudocoelom into the germline.

2) Regarding the roles of *ent-1* and *ent-2* in development, we observed that the CRISPR-generated complete double knockout mutant (*ent-1 KO; ent-2 KO*) exhibits severe developmental delays, arrest, and sterility, making it not feasible to maintain the strain. However, the double loss-of-function models mainly used in this study (*ent-1 KO* with *ent-2 RNAi* knockdown and *ent-2 KO* with *ent-1 RNAi* knockdown) can develop to the adult stage but exhibit sterility. Therefore, *ent-1* and *ent-2* do play crucial roles in development. The relatively milder developmental phenotypes observed in the double loss-of-function models than the complete double knockout mutant suggest that reproduction may be more sensitive to guanosine dysregulation than development.

3) As suggested by Reviewer 2, we have also included a new discussion regarding the role of pyrimidine in regulating reproduction: Previous studies have demonstrated that dietary pyrimidine nucleotide U/T level is critical for maintaining germline proliferation in *C. elegans* through the GLP-1/Notch receptor, a key regulator of germline proliferation and the mitosis-to-meiosis transition (PMID: 26833730). The intestinal nuclease ENDU-2 has also been reported to regulate nucleotide balance and germline proliferation (PMID: 32049015). Together with our new findings, these studies revealed that the transport and interplay of nucleosides and nucleotides between somatic tissues and the germline is a crucial mechanism in regulating reproduction.

4) We have discussed the ENT family transporter in other models of reproduction: In higher eukaryotes, members of the human ENT family, such as hENT1 and hENT2, are highly expressed in both the digestive and reproductive systems, as reported in the Human Protein Atlas (proteinatlas.org) (PMID: 25613900). This suggests that cross-tissue coordination of nucleoside transport may similarly play a role in reproduction in more complex organisms.

4. Line 214: “conduced” should be “conducted”?

Response: We have corrected this error. Thank the reviewer for pointing this out.

Reviewer #2: Here, Guan, et al. report that they found that knockdown of ent-1 or ent-1 slight reduces total progeny number, and the double reduction has a larger effect on reproduction. GFP tagging revealed tissue specificity of ENT-1 and -2, in germline and intestine vs gonad and intestine, respectively. Degradation of the ENTs in specific tissues suggested that ENT-1 in germline and ENT-2 in intestine function to regulate reproduction. Single-nuc seq analysis suggests that the double mutants have fewer proliferating germ cells, and that (surprisingly?) purine metabolism genes are upregulated in the germline. LC/MS showed that there are differences in levels of G, A, and I , but not C, between controls and the double mutants. Microinjection of G into the pseudocoelom at L4 increased brood size, but A did not.

Response: We thank the reviewer for her/his constructive comments and suggestions to further strengthen our manuscript.

1. What is the percent effect of progeny number reduction? It looks extremely slight (Fig. 1C, 1D). Please report this % in the text and in the figure legend.

Response: We thank the reviewer for the suggestion. We have included the percentage information in the text and figure legend.

2. The terminology isn't that it "increased" progeny in the later reproduction – this could just be slowing of egg-laying.

Response: We thank the reviewer for the suggestion. We have changed the terminology to make the statement more precise.

3. (Out of curiosity - How does one propagate a CRISPR strain with no progeny production?)

Response: We appreciate the reviewer's interest in this point. Our data indicated that the single CRISPR knockout mutant of *ent-1* or *ent-2* exhibits minimal or no reproductive phenotypes. However, when we crossed the single mutants to generate a double knockout mutant (*ent-1 KO; ent-2 KO*), the resulting strain was completely sterile and displayed developmental delays and vulva protrusion. These findings suggest that *ent-1* and *ent-2* function synergistically to regulate reproduction. Unfortunately, the sterility of the double knockout strain prevented its propagation. To address this, we utilized the double loss-of-function models that carry RNAi-mediated knockdown of *ent-2* in the *ent-1* knockout background (*ent-1 KO; ent-2 KD*) or ent-1 RNAi knockdown in the ent-2 knockout background (*ent-2 KO; ent-1 KD*). In both conditions, the animals can reach adulthood even though brood sizes were significantly reduced.

4. I understand that the authors have previously published on single-nucleus sequencing, but why is that a relevant experiment here, if the authors already showed where the proteins function and that the relevant changes are in the germline to regulate reproduction? (How would this be different than whole-worm analyses? Were there actually differences between single cells that would have been invisible in whole-worm analyses?) The logic is unclear.

Response: We thank the reviewer for raising this question. We agree with the reviewer that genetic evidence using tissue-specific rescue and knockdown had helped us demonstrate the functional tissues of ENT-1 and ENT-2 in regulating reproduction. We expanded our studies to single-nucleus RNA-seq analyses, because we aim to understand tissue-specific transcriptome changes in response to the loss of ENT-1 and ENT-2. Through these analyses, we discovered germline-specific up-regulation of both *de novo* and *salvage* biosynthesis pathways of purine but not pyrimidine nucleotides. However, in the intestine, the *de novo* biosynthesis pathway of purine nucleotides is downregulated. No significant changes were detected in other tissues, like

the hypodermis and muscle. Those tissue-specific transcriptional changes cannot be resolved using whole-worm RNA-seq analyses.

5. What would the single mutant snSeq show in specific tissues? This would go far to support the hypothesis they later state about the intestinal role.

Response: We thank the reviewer for this insightful suggestion. In single-knockout mutants, reproductive phenotypes were similar to wild-type animals. Consistently, we did not observe significant changes in the mRNA expression of purine metabolism-related genes in either the germline or intestine of the single knockout mutant (**Fig 3 H, I, S3 Fig B, C**).

6. The pseudo-time analysis is nice and should be in the main figures.

Response: We appreciate the reviewer's suggestion and have included the pseudo-time analysis in the main figures.

7. It looks like there are two distinct populations in the guanosine microinjections – do the authors know what might have led to this huge difference in brood size (<50 vs >200)?

Response: We thank the reviewer for pointing out this. In this experiment, nucleoside or control solutions were injected into the limited pseudocoelom space of mid-L4 larvae. We chose this time point because performing microinjections at earlier stages is technically challenging due to the larvae's smaller size. However, injecting at this stage limits the time window for achieving full rescue before fertilization and egg-laying. While we kept microinjection duration and pressure consistent across replicates, variations in the volume of the solution injected into the pseudocoelom may arise from differences in needle opening size and uncontrollable variations in pseudocoelom resistance. Together with differences in pseudocoelom volume among individual worms, these factors contribute to variations in final nucleoside concentration, which likely explain the observed variability in the results. In addition, although we carefully synchronized worms, mid-L4 larvae may vary slightly in their developmental timing. As a result, worms that receive the correct concentration of injected nucleosides earlier in development are more likely to show near-complete rescue, while others may exhibit minimal or no rescue.

8. In Figure 4F, how did the authors come to their conclusions in this table? Are these data? If so, from what experiment did these significance (or whatever the +++ means) come from? Or is this a model? If the latter, it needs to be much clearer in the text, and perhaps depicted as a model rather than as if it is showing data (but if the latter, the data need to be clearer).

Response: We thank the reviewer for their comment. The table in Figure 4F represents a predicted model illustrating the relative guanosine levels in different tissues of the single and double mutants. To avoid confusion, we have clarified in the text and figure legend that this is a conceptual model rather than a direct representation of raw data, as suggested by the

reviewer. We have also changed “Guanosine Level” to “Predicted Guanosine Level” in the figure panel.

We proposed this model based on our experimental results, which suggest that ENT-1 and ENT-2 function in the germline and intestine, respectively, to coordinate reproduction. ENT-1 and ENT-2 are both passive transporters that maintain nucleoside equilibrium between two compartments. In the *ent-1KO* single-knockout mutant, the equilibrium between the pseudocoelom and germline is disrupted, while the equilibrium between the intestine and pseudocoelom remains intact. Conversely, in the *ent-2KO* single-knockout mutant, the equilibrium between the intestine and pseudocoelom is disrupted, but the equilibrium between the pseudocoelom and germline is preserved. In both cases, the guanosine nucleoside level in the germline is reduced yet it remains sufficient to prevent sterility. In the *ent-1KO; ent-2KD* double loss-of-function worms, however, disruptions in both intestinal export and germline import result in a severe reduction in germline guanosine levels, leading to sterility. Microinjection of guanosine increases its levels in the pseudocoelom, which can partially rescue the sterile phenotype. We hope the presentation of this integrated model helps readers better understand our findings and the underlying mechanisms.

9. How does the current work fit into the context of previously known work from Min Han’s lab on the role of nucleotides and nucleotide metabolism and nucleotide-sensing mechanisms for controlling reproduction (Jia et al. 2020; Chi et al. 2016)?

Response: We appreciate that the reviewer has brought these prior findings into our attention. Han’s work elucidated that the cytidine deaminases *cdd-1/2* double knockout worms with impaired U/T synthesis are sterile when growing on the OP50 *E. coli* strain that provides limited U/T (PMID: 26833730). It also showed that GLP-1 expression is modulated by dietary U/T levels, linking dietary nucleotide availability to germ cell proliferation. Additional findings further demonstrated that the endonuclease ENDU-2 in the intestine is induced by nucleotide imbalance and consequently regulates germ cell proliferation via affecting the phosphorylation of CTP synthase CTPS-1 (PMID: 32049015). These studies reveal how the pyrimidine nucleotides signal through their sensing factors to regulate *C. elegans* reproduction.

Our work instead focuses on the direct nucleoside transport mechanism between somatic tissues and the germline and its crucial roles in regulating production. For the two characterized ENT-1 and ENT-2 transporters, we uncovered their predominant activities in the transport of guanosine nucleosides between the intestine and germline. The sterile phenotype in the double knockout mutants indicates that germline guanosine availability is essential for successful reproduction. We believe these studies together support the importance of intestinal nucleosides/nucleotides to sustain fertility. We have included additional discussions regarding the Han’s work.

Minor:

- Change “Our recent findings found” to “We recently found”

- seems excessive to claim this as a “pioneering” example
- not “in human” but “in humans”

Response: We have revised these accordingly. Thank the reviewer for pointing them out.

Reviewer #3: This manuscript by Guan and colleagues details the intricate trafficking of nucleosides and nucleotides between somatic and germline tissues via the SLC29A family of transporters using C. elegans as a system. The authors identify a pair of equilibrative nucleoside transporters ENT-2 and ENT-1 which act in soma and germline respectively to fulfill organismal nucleoside needs for reproduction. Loss of ent-1 and ent-2 prompts synthetic lethality, indicating cooperative roles in nucleoside and nucleotide homeostasis in the intact organism. The authors go on to use elegant and overlapping lines of evidence to uncover tissue-specific roles of each transporter. The use of snRNAseq illuminates tissue specific transcriptional responses to the loss of either or both ENT genes, and metabolomics subsequently elucidates precisely the nucleosdies altered with ENT loss. The methods used are rigorous and results clearly presented. I do have some concerns with the statistics used and failure to clearly indicate numbers of biological replicates, however I remain enthusiastic overall for the novelty and findings presented by the authors herein. Also, some alternative possibilities exist for why there is synthetic lethality with dual ent1/2 loss, and these should be explored and/or discussed.

Response: We thank the reviewer for their kind words and for being enthusiastic about our paper as well-written and novel.

MAJOR:

1. Are deletion mutants available for the ent genes, and if so, are they viable? Over-reliance on RNAi in the initial ENT gene survey might mask deleterious effects of loss of ent genes other than ent-1 and ent-2.

Response: We thank the reviewer for the suggestion. In response, we have included additional insights regarding the potential roles of other *ent* genes in reproduction. Since RNAi used in the initial ENT gene screening might have masked deleterious effects of *ent* genes beyond *ent-1* and *ent-2*, we measured brood sizes in mutants of *ent-3 (ok945)*, *ent-4(ok2161)*, *ent-6 (syb7180)*, and *ent-7 (ok1238)* (noting that an *ent-5* mutant is currently unavailable). Our analysis revealed that the *ent-4* deletion mutant displayed a significantly reduced brood size compared to the wild type worms (**S1 FigJ**).

Interestingly, prior research (PMID: 38991033) indicates that ENT-4 is specifically localized to the apical membrane of intestinal cells, suggesting that it may mediate the uptake of nucleosides from the diet to regulate reproduction. In contrast, ENT-2 localizes to the basolateral side of the intestine, indicating a distinct role in exporting nucleosides from the intestine to the pseudocoelom. Taken together, *ent* family genes might support reproduction in a coordinated model: ENT-4 facilitates dietary nucleoside uptake into the intestine, ENT-2

exports nucleosides from the intestine to the pseudocoelom, and ENT-1 imports nucleosides from the pseudocoelom into the germline.

2. This reader sincerely appreciates the inclusion of detailed statistical information on sample size in each figure legend. None-the-less, several of the statistics are improperly applied (e.g. t-tests without multiple hypothesis testing as in multiple comparisons in Fig. 1E), and it remains unclear from the data presented how many times the experiment was repeated, and whether these “n” are from one biological replicate or whether the experiment was sufficiently replicated.

Response: We thank the reviewer for highlighting this issue. The analysis for Figure 1E was conducted using a one-way ANOVA with Holm–Sidak correction but was incorrectly labeled in the figure legend. This has now been corrected. Most experiments in this study were performed with at least three independent biological replicates. The "n" values reported in the manuscript represent the total number of animals across all biological replicates. We have updated the figure legends to provide this information more clearly.

3. T-tests were again inappropriately applied when two-way ANOVA or similar with post-hoc correction should have been used for Figures 2C, D, E and one-way ANOVA or similar for figures 4G-H.

Response: We thank the reviewer for pointing this out. We acknowledge that T-tests were not the appropriate statistical method for Figures 2C, D, E, and 4G–H. For Figures 2C, D, and E, we have reanalyzed the data using two-way ANOVA with post-hoc correction. Similarly, for Figures 4G and 4H, we have reanalyzed the data using one-way ANOVA with post-hoc correction.

4. A possibility remains that the synthetic lethality in double ent-1 and ent-2 KO is because of combined action in germline (or intestine). While this is unlikely, the authors have the ability to test combined AID of both ENT-1 and ENT-2 in germline and intestine. This dual loss would support their model quite convincingly beyond the add-back and single conditional AID experiments. Also it could explain why there is no visible drop in fecundity in spite of reduced guanosine levels (Fig. 4) if the more severe drop with double loss was exhibited in specific tissues which are not resolvable by whole animal LC-MS. If the authors do not think this strengthens their story they should at least indicate why they believe this experiment to be unnecessary.

Response: We appreciate the reviewer’s suggestion and agree that simultaneously knocking down ENT-1 in the germline and ENT-2 in the intestine would strengthen our findings. However, this cannot be achieved by combining AID. If we crossed the four strains: *degron-ent-1*, *degron-ent-2* strains, the strains expressing TIR1 under tissue-specific promoters: *ges-1p-TIR1* for the intestine, and *gld-1p-TIR1* for the germline, the auxin treatment in the crossed line would result in the degradation of both ENT-1 and ENT-2 in both tissues rather than the desired tissue-specific knockdown, as both *ent-1* and *ent-2* are endogenously tagged with the AID sequence. Given that the ENT-2 protein is endogenously expressed in the intestine and somatic gonad but

not in the germline, the strain combining *ent-2* RNAi knockdown, *gld-1p-TIR1*, and *degron-ent-1* upon the auxin treatment will lose ENT-1 in the germline and ENT-2 in the intestine. As shown in **Fig 2G**, this strain exhibited significantly reduced progeny compared to the single mutants, supporting the proposed model.

5. Why are Adenosine and inosine rising so dramatically in the single ent and double ent loss? Is the drop in gmps-1 expression in the salvage pathway partly to blame? The authors should explain why they are seeing these dramatic shifts away from G towards A/I.

Response: We appreciate the reviewer's insightful comments and questions. It is indeed an interesting hypothesis that the downregulation of *gmps-1* contributes to the elevated adenosine and inosine levels. However, we note that *gmps-1* is not downregulated in the *ent-1* or *ent-2* single mutant, despite the observed increase in adenosine and inosine levels.

Here is our hypothesis to explain these changes: ENT-1 and ENT-2 play critical roles in transporting various purine nucleosides, including guanosine, adenosine, and inosine, from the intestine to the germline. In the germline, successful DNA and RNA synthesis for reproduction requires sufficient levels of dATP/ATP and dGTP/GTP. While dGTP/GTP levels in the germline are largely dependent on guanosine transport from the intestine, dATP/ATP is primarily derived from other sources such as mitochondrial oxidative phosphorylation and is less reliant on adenosine/inosine. In the *ent* single and double mutants, germline DNA and RNA synthesis likely reduces the guanosine pool, while having little impact on the adenosine/inosine pool. This reduction in germline guanosine levels may trigger a compensatory upregulation of overall purine biosynthesis, which could account for the observed increases in adenosine and inosine levels. However, despite the compensation, guanosine levels remain lower due to its high demand for DNA and RNA synthesis. In the double mutant, the upregulation of both the *de novo* and *salvage* purine metabolism pathways is evident at the transcriptomic level, directly supporting our hypothesis. On the other hand, in the single mutants, the compensation likely occurs at the protein level or through elevating enzymatic activity, which are beyond the resolution of snRNA-seq analysis. We have incorporated these discussions into the revised manuscript.

6. While I think the model is compelling, alternative explanations exist, and unless the authors invoke non-transporter mediated G uptake in germline in the microinjection experiments in 4G, we can surmise that additional SLCs participate in uptake of G into germline or compensation for loss of ent-1ko. Could dual ent-1 and ent-2 roles in the germline be an alternate explanation? There are other possibilities as well which the authors should at least briefly discuss and/or test if easy to do so.

Response: We thank the reviewer for the insightful comments and have included additional discussions in the manuscript regarding alternative explanations.

Our findings suggest that *ent-1KO* or *ent-2KO* single-knockout mutants do not exhibit reproductive phenotypes, and microinjection of guanosine into the pseudocoelom of the *ent-*

1/ent-2 double loss-of-function worms restores fertility. These observations indicate the presence of compensatory mechanisms beyond ENT-2's export role in the intestine and ENT-1's import role in the germline that contribute to the maintenance of guanosine levels required for reproduction. Combining the tissue-specific rescue/knockdown results and protein expression patterns — in which ENT-1 is expressed in the intestine and germline, and ENT-2 is expressed in the intestine and somatic gonad—it is less likely that ENT-2 works together with ENT-1 in the germline because ENT-2 is not expressed there.

It is plausible that other SLC transporters, including additional ENT family members, contribute to guanosine transport in the intestine and germline, albeit with lower affinity or efficiency. Furthermore, concentrative nucleoside transporters (CNTs), which leverage ion gradients to actively transport nucleosides against their concentration gradients, may also play a compensatory role. We propose that ENT-1/ENT-2-mediated guanosine transport follows a gradient from the intestine (highest concentration) to the germline (lowest concentration) via the pseudocoelom. When this concentration gradient is disrupted by the loss of ENT-1 and ENT-2, CNTs may compensate by actively transporting guanosine from the intestine to the germline. In the single *ent-1* or *ent-2* mutant background, only one aspect of guanosine transport—either export from the intestine or import into the germline—is impaired. Under these conditions, the less efficient compensatory transport mechanisms appear sufficient to sustain reproduction, although changes in nucleoside levels are already detectable via MS. However, in the double mutant background, where guanosine levels are critically low, these compensatory mechanisms are inadequate. Compensation may also occur through the upregulation of guanosine synthesis via the *de novo* and *salvage* pathways from multiple substrates, such as amino acids, as illustrated by snRNA-seq analysis in our study. Furthermore, while we focused on guanosine transport from the intestine to the germline, it is conceivable that the germline obtains guanosine from other tissues, such as muscle or hypodermis, through other nucleoside transporters expressed in these tissues. Investigating nucleoside transport and metabolism in these alternative tissues would be an intriguing aspect for future research.

MINOR:
There are a few minor grammatical errors, e.g. ln 34 "collectively termed as metabolites." Should probably be "collectively termed metabolites…"
Ln133 "exon sequences" should probably specify "coding exonic nucleotide sequence" or similar.
"Offsprings" in Y-axis labels should probably be "offspring"

Response: We have revised these accordingly and thank the reviewer.

It appears that brood is already reduced modestly in the -Auxin AID strains, suggestive of either a negative pleiotropy of tagging or that there is modest constitutive reduction in ENT expression. Either way these possibilities are informative for the field and should at least be mentioned in the manuscript for those less experienced in making use of the AID system in vivo.

Response: We thank the reviewer for raising this point and have now included this information in the revised manuscript. When generating the AID strains, we have included a necessary linker between the degron sequence and ENT-1/2 to prevent disruption of the transporter structures. Despite this precaution, we observed that tagging AID to the N-terminus of *ent-1* and *ent-2* partially compromises their functions, as evidenced by reduced brood sizes in the endogenous AID-tagged strains under the mutant background of the other *ent* gene. We also attempted C-terminal AID tagging for both transporters and found these strains displayed even greater functional disruption, with *ent-1::degron; ent-2KO* and *ent-1KO; ent-2::degron* phenocopied the severe defects in the complete double knockout mutant, including developmental delays and sterility.

Although previous studies (PMID: 26552885) have demonstrated that TIR1 expression and auxin treatment generally do not affect viability, fertility, or development, and the degron sequence is relatively small (44 amino acids) as a tag, our findings highlight the importance of carefully selecting the degron-tagging site in transmembrane proteins. These observations should serve as a valuable reference for researchers utilizing the AID system to tag transmembrane transporters in vivo.
