# Supplementary material for: Cross-tissue coordination between SLC nucleoside transporters regulates reproduction in Caenorhabditis elegans
Source: PLoS Genet. 2025 May 30;21(5):e1011425. doi: 10.1371/journal.pgen.1011425 (PMC12151481; doi:10.1371/journal.pgen.1011425)
Supplement: Attachment — Submitted filename: Point-by-point.pdf [file pgen.1011425.s006.pdf]

Reviewer #1: The authors have very comprehensively addressed all reviewer concerns. I highly recommend the manuscript for publication.

Reviewer #3: The authors have answered all of my concerns. The manuscript is well conducted and rigorously supported.

Response: Thank the reviewers for recommending our manuscript for publication.

Reviewer #2: While the authors have addressed most of my comments, the table in Figure 4F continues to be misleading and should be removed entirely, as it is too easily misconstrued as data when in fact it is not. I realize that the authors have added "Predicted [sic]" to the top of the table, but again, it is not appropriate to depict this model as a table. The authors should figure out how to present their model in a way that does not suggest that they have actually made these measurements of guanosine in tissues, which they have not done - it is too easy for readers to mistake this table as data, and believe that the authors have somehow developed tissue-specific measurements of nucleotides. Instead, they should make a graphical figure of their predictions. I'm sorry that this is extra work, but it is dangerous to present models in formats normally used for data.

Response: We appreciate the reviewer's comment and have removed the table to avoid any potential confusion.
